# *Salvia miltiorrhiza* Injection Alleviates LPS-Induced Acute Lung Injury by Adjusting the Balance of MMPs/TIMPs Ratio

**DOI:** 10.1155/2020/9617081

**Published:** 2020-07-20

**Authors:** Guobing Chen, Dandan Ge, Bizhen Zhu, Huixuan Shi, Qilin Ma

**Affiliations:** ^1^Department of Paediatrics, The First Affiliated Hospital of Xiamen University, Xiamen 361003, China; ^2^School of Medicine, Xiamen University, Xiamen 361003, China; ^3^Pediatrics Key Laboratory of Xiamen, Xiamen 361003, China; ^4^Department of Neurology, The First Affiliated Hospital of Xiamen University, Xiamen 361003, China

## Abstract

*Salvia miltiorrhiza* injection (SMI) is a classical traditional Chinese medicine, which plays an active role in the treatment of many diseases such as promoting blood circulation, removing blood stasis, reducing inflammatory reaction, and improving acute lung injury (ALI). Previous studies have shown that matrix metalloproteinases (MMPs) and tissue inhibitors of metalloproteinases (TIMPs) are involved in the pathophysiological process of ALI. However, the relationship between SMI and MMPs/TIMPs remains unclear. In this study, Wistar rats were randomly divided into control group (NC), *Salvia miltiorrhiza* group (SM), lipopolysaccharide group (LPS), and *Salvia miltiorrhiza* treatment group (Tsm). The four groups were subdivided into four time points (2, 6, 12, and 24 hours), and specimens were collected after animal sacrifice at each time point. Serum TNF-*α* and IL-6 levels were detected by ELISA. The degree of lung injury was determined by lung tissue hematoxylin-eosin staining, lung wet/dry weight (W/D) ratio, and lung permeability index. The changes in lung MMPs/TIMPs protein and mRNA were detected by Western blot and real-time quantitative PCR. The results showed that rats injected with LPS experience acute lung injury, and the ratio of MMPs/TIMPs in lung tissues increased gradually with time. In the Tsm group, the ratio of MMPs/TIMPs decreased gradually, and likewise, the balance was gradually restored, while indicators related to lung injury were gradually declined. These data suggest that SMI alleviates LPS-induced acute lung injury; this protective effect may be related to regulation of the balance of MMPs/TIMPs ratio.

## 1. Introduction

Acute lung injury (ALI) is a common critical illness in clinic that can manifest as severe hypoxia and dyspnea, has a relatively high mortality rate, and is difficult to treat [1, 2]. The pathogenesis of ALI has not been fully elucidated, but the inflammatory response caused by the release of LPS from bacterial infection is believed to be the main cause [3, 4]. ALI is a fatal disease characterized by inflammatory cell infiltration, alveolar-capillary barrier disruption, protein-rich pulmonary edema, and impairment of gas exchange [5, 6]. Alveolar-capillary membrane injury is the main cause of ALI [7, 8].

The extracellular matrix (ECM) is the main structural part of the alveolar-capillary membrane, which not only plays a mechanical-supporting and mechanical-connecting role between cells but is also important in maintaining normal tissue structure and function [9, 10]. Studies have shown that matrix metalloproteinases (MMPs) and tissue inhibitors of metalloproteinases (TIMPs) are the most important enzyme systems involved in ECM metabolism [11, 12]. Under normal circumstances, if the lung MMPs/TIMPs ratio is balanced, synthesis and degradation of the ECM are in a state of dynamic equilibrium [12, 13]. Therefore, it is reasonable that the balance of the MMPs/TIMPs ratio is key to lung tissue injury and repair.

*Salvia miltiorrhiza* injection (SMI), a Chinese Materia Medica standardized product extracted from the root of red-rooted Salviae Miltiorrhizae (*Salvia miltiorrhiza* Bunge, Labiatae, Danshen in Chinese), which is officially recorded in Pharmacopoeia of the People's Republic of China (version 2010) and plays an active role in the treatment of many diseases such as promoting blood circulation, removing blood stasis, reducing inflammatory reaction, and improving coagulation [14].

Previous studies have shown that SMI has a protective effect on ALI [14, 15], but the mechanism remains unclear. Therefore, we sought to answer the following question: does SMI provide a lung protection effect by restoring the balance of the MMPs/TIMPs ratio?

## 2. Materials and Methods

### 2.1. *Salvia miltiorrhiza* Injection (SMI)

*Salvia miltiorrhiza* injection (SMI) was purchased from Chiatai Qingchunbao Pharmaceutical Co., Ltd. (Chinese medicine quasiword z33020177, Zhejiang, China). It is a classical traditional Chinese medicine standardized aqueous product extracted from the root of red-rooted *Salvia* (scientific name: *Salvia miltiorrhiza* Bunge, perennial erect herb of the genus Dicotyledonaceae, Danshen in Chinese). SMI is an injectable solution through modern formulation and preparation process; the main ingredient is tanshinone, which is officially recorded in Pharmacopoeia of the People's Republic of China (version 2010) and plays an active role in the treatment of many diseases [14].

### 2.2. Animals

Specific pathogen-free (SPF) male Wistar rats (2 months old, 180 ± 200 g) were purchased from Charles River Experimental Animal Co., Ltd. (Beijing, China) and raised in the Xiamen University Laboratory Animal Center (License number: SYXK (Min) 2018-0010). The animals were housed one week before the start of experiment under standard laboratory conditions at a stable temperature (22 ± 2 °C), humidity (50 ± 10%), and a 12/12 h light/dark cycle with food and water ad libitum [15, 16]. All animal procedures were carried out at the Xiamen University Laboratory Animal Center and approved by the Ethics Committee of the First Affiliated Hospital of Xiamen University.

### 2.3. Animal Modeling and Grouping

In total, 96 Wistar rats were randomly divided into a normal control (NC) group, *Salvia miltiorrhiza* (SM) group, lipopolysaccharide (LPS, Sigma-Aldrich, St. Louis, MO, USA, cat. No. L2880) group, and *Salvia miltiorrhiza* treatment group (LPS + SM: Tsm group). In the LPS group, an experimental model of ALI was established by intravenous injection of 5 mg/kg LPS [17]. The SM group was injected with 50 mg/kg *Salvia miltiorrhiza* injection. The Tsm group was given 50 mg/kg *Salvia miltiorrhiza* injection one hour after 5 mg/kg LPS injection. The NC group was injected with 2 mL/kg normal saline as a control. The four groups were further divided into 2, 6, 12, and 24 hours subgroups with six rats at each time point. The rats were anesthetized via intraperitoneal injection with 2.25% pentobarbital sodium (45 mg/kg, WS 20060401, Sinopharm Chemical, China) [18], and blood, lung tissue and bronchoalveolar lavage fluid (BALF) specimens were collected at each designated time point. The research was conducted in accordance with the internationally accepted principles for laboratory animal use and care.

### 2.4. Enzyme-Linked Immunosorbent Assay (ELISA)

The rats were anesthetized, followed by collecting 3 mL of blood from the abdominal aorta. After centrifuging the blood samples at 3,500 g for 10 min, the supernatants were collected. TNF-*α* (Quantikine, cat. no. RTA00) and IL-6 (Abclonal, cat. no. RK00020) levels were measured according to the ELISA procedures as described by the manufacturers, in order to investigate the inflammatory reaction in rats.

### 2.5. Hematoxylin-Eosin (H&E) Staining

Small pieces of upper left lung tissue were fixed in 4% formaldehyde for 24 h, followed by conventional paraffin embedding and tissue sectioning at 5 *μ*m thickness [19]. The tissue sections were stained with H&E (Solarbio) and the morphological changes of the lung tissues were observed under a light microscope to understand the degree of lung injury.

### 2.6. Lung Wet/Dry Weight (W/D) Ratio

The wet/dry weight ratio is an index of pulmonary edema. The inferior lobe of the left lung was excised and the wet weight was recorded before being placed in an incubator at 60°C for 72 h to obtain the dry weight [14]. The lung wet/dry weight ratio was calculated for each group.

### 2.7. Detection of Pulmonary Permeability Index

Detection of sample protein was performed by bicinchoninic acid (BCA) protein quantitative kit (Tiangen Biotech Co., Ltd., cat. no. PP0101, Beijing, China). The absorbance was measured at 562 nm wavelength with a multifunction microplate reader, and a protein standard curve was drawn. A tracheoalveolar lavage was performed 3 times using 1 ml phosphate buffered saline (PBS) per lavage on the upper right bronchi and the bronchoalveolar lavage fluid (BALF) was collected (90% recovery). The protein contents of BALF and plasma were calculated according to the absorbance values. To determine the extent of the lung injury, the pulmonary permeability index (PPI) was calculated using the following equation: PPI = BALF protein content/plasma protein content [20].

### 2.8. Real-Time Quantitative PCR Analysis

Approximately 50 mg of right middle lobe lung tissue was used for total RNA extraction (Promega, cat. no. LS1040) using the TRIzol (Tiangen Biotech Co., Ltd., cat. no. DP405-02, Beijing, China) method [21]. After analyzing the total RNA concentration and purity, *β*-actin was used as an internal control for quantitative real-time PCR (qPCR, Promega, cat. no. A6002) detection of MMPs and TIMPs. cDNA synthesis kit was purchased from Tiangen Biotech Co., Ltd. (cat. no. KR116-02, Beijing, China). The forward and reverse primer sequences (Sangon Biotech Co., Ltd., Shanghai, China) for MMP2, MMP9, TIMP1, TIMP2, and *β*-actin are shown in Table 1. The qPCR conditions were preliminary denaturation 95°C for 2 min, followed by 40 cycles of denaturation at 95°C for 15 s, annealing at 58°C for 34 s, and elongation at 72°C for 1 min, followed by a final elongation step at 72°C for 5 min, performed on a CFX96 Touch Real-Time PCR Detection System (BioRad, Hercules, CA, USA). PCR products were separated on 1% agarose gels and visualized using ethidium bromide staining and UV light to verify the products sizes. Glyceraldehyde 3-phosphate dehydrogenase was used as the loading control for normalization of the data. Data from qPCR were analyzed using the 2^–ΔΔCt^ method [22].

### 2.9. Western Blot Analysis

Approximately 50 mg lower right lung tissue samples were collected from the different time point groups of rats, followed by washing, homogenization, and lysing [23]. After conventional protein extraction from the lung tissues, the total protein concentration was measured, followed by adding 30 *μ*g total protein to 1/3 volume of 4 × SDS-PAGE (Sinopharm Chemical Reagent Co., Ltd., cat. no. 30166428) sample buffer, boiling the protein sample for 10 min, and separating the proteins by SDS-PAGE. After transferring the separated proteins onto PVDF membrane (Emd Millipore BioTechniques Co., Ltd., cat. no. IPVH00010), the protein blots were prepared according to the conventional procedures of Western blot analysis [24]. After developing the protein blots in ECL (Advansta, cat. no. K-12043-D10), the absorbance values of protein bands were scanned by an Odyssey infrared gel imaging system (LI-COR, Lincoln, NE, USA) to calculate the relative protein expression levels of MMPs and TIMPs according to the ratios of MMPs and TIMPs absorbance to *β*-actin absorbance. Related antibodies and companies required in Western blot analysis are shown in Table 2.

### 2.10. Statistical Analysis

All statistical analyses were performed with SPSS 21.0 software (IBM SPSS, Chicago, IL, USA). Each measurement is presented as mean ± SD ($\bar{x}$ ± *s*). The independent sample *t*-test was used for comparison between groups. One-way ANOVA was used for comparison within groups. Pearson's correlation analysis was used for the relevant trend variables. *p* < 0.05 was considered statistically significant.

## 3. Results

### 3.1. Changes in the Serum Inflammatory Factors TNF-*α* and IL-6

**Figure 1:**
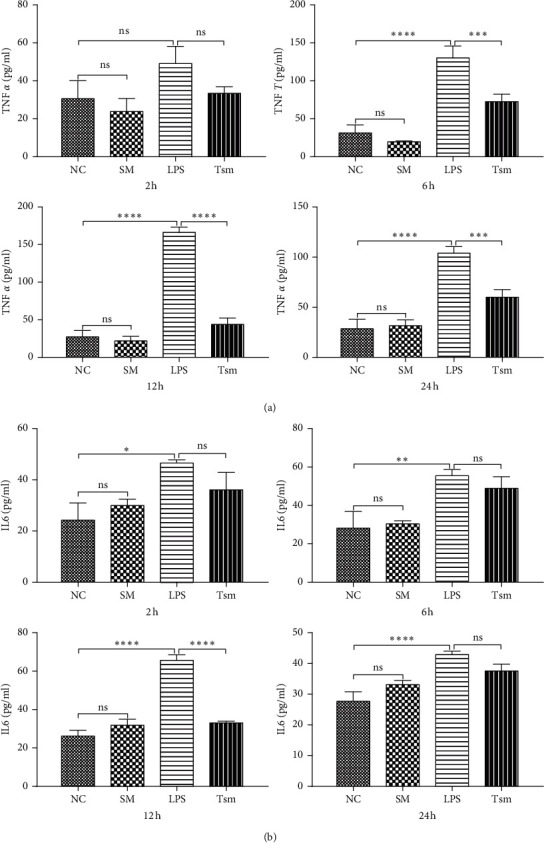
TNF-*α* and IL-6 changes in each group. (a) Changes in TNF-*α* content. (b) Changes in IL-6 content. NC, normal control group; SM, *Salvia miltiorrhiza* group; LPS, lipopolysaccharide group; Tsm, *Salvia miltiorrhiza* treatment group. ns, no statistical difference. 2 h, 6 h, 12 h, and 24 h indicate 2, 6, 12, and 24 hours' time points, respectively. Data are shown as mean ± SD (*n* = 6 per group). ^*∗*^*p* < 0.05; ^*∗∗*^and ^*∗∗∗*^*p* < 0.01; ^*∗∗∗∗*^*p* < 0.0001.

Compared with the NC group, the values of TNF-*α* and IL-6 in the LPS group increased with time; the changes of TNF-*α* had significant increases at the other three time points, except 2 hours (Figure 1(a)), and the changes of IL-6 had significant increases at all four time points (Figure 1(b)).

In the Tsm group, the values of TNF-*α* and IL-6 decreased by varying degrees with time compared to the LPS group: TNF-*α* was significantly decreased at 6, 12, and 24 hours' time points (Figure 1(a)); and IL-6 was significantly decreased at the 12 hours' time point (Figure 1(b)). There was no significant difference between the NC group and SM group across the four time points (Figure 1).

### 3.2. Changes in Indicators Related to Acute Lung Injury

**Figure 2:**
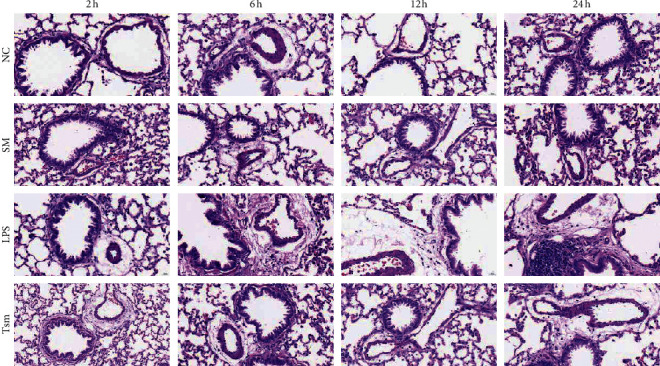
Histopathology changes in lung by H&E staining (400× magnification). NC, normal control group; SM, *Salvia miltiorrhiza* group; LPS, lipopolysaccharide group; Tsm, *Salvia miltiorrhiza* treatment group. 2 h, 6 h, 12 h, and 24 h indicate 2, 6, 12, and 24 hours' time points, respectively.

Pathological sections of lung tissues showed swollen alveolar epithelial cells and infiltrated inflammatory cells after LPS injection. However, the pathology of lung injury was gradually alleviated in the Tsm group. The structure of the bronchus, alveoli, and blood vessels in the NC group and SM group were normal (Figure 2).

**Figure 3:**
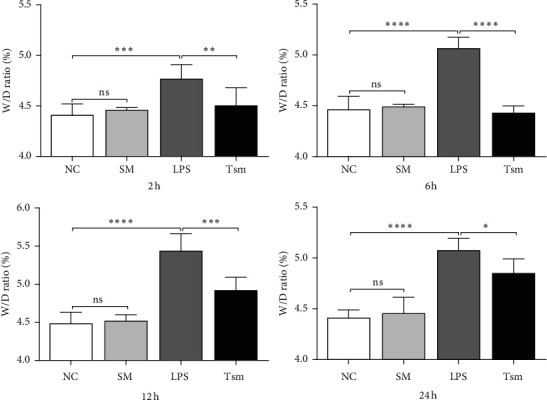
Lung wet/dry (W/D) ratio in each group. NC, normal control group; SM, *Salvia miltiorrhiza* group; LPS, lipopolysaccharide group; Tsm, *Salvia miltiorrhiza* treatment group. ns, no statistical difference. 2 h, 6 h, 12 h, and 24 h indicate 2, 6, 12, and 24 hours' time points, respectively. Data are shown as mean ± SD (*n* = 6 per group). ^*∗*^*p* < 0.05; ^*∗∗*^and ^*∗∗∗*^*p* < 0.01; ^*∗∗∗∗*^*p* < 0.0001.

**Figure 4:**
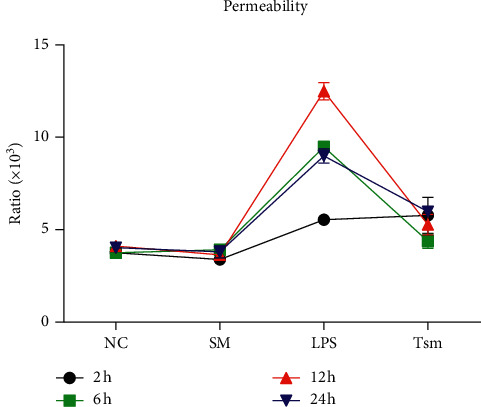
Lung permeability changes in each group. NC, normal control group; SM, *Salvia miltiorrhiza* group; LPS, lipopolysaccharide group; Tsm, *Salvia miltiorrhiza* treatment group. 2 h, 6 h, 12 h, and 24 h indicate 2, 6, 12, and 24 hours' time points, respectively.

Compared with the NC group, the lung W/D ratio and lung permeability index in the LPS group were increased significantly at all time points. However, compared with the LPS group, the above indicators in the Tsm group decreased gradually with time (Figures 3 and 4).

### 3.3. Changes in Lung MMPs, TIMPs mRNA, and Protein Expression

**Figure 5:**
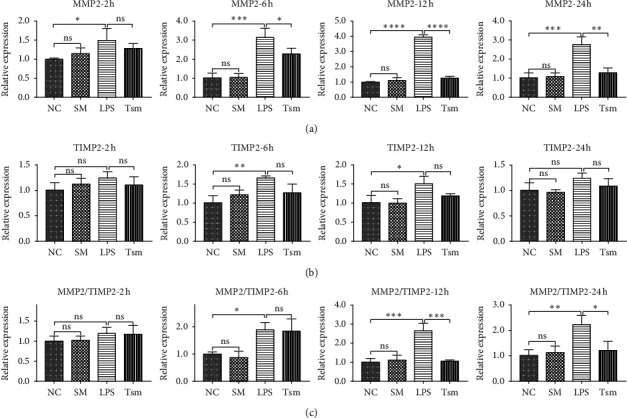
(a) MMP2 mRNA expression; (b) TIMP2 mRNA expression; (c) MMP2/TIMP2 ratio. NC, normal control group; SM, *Salvia miltiorrhiza* group; LPS, lipopolysaccharide group; Tsm, *Salvia miltiorrhiza* treatment group. ns, no statistical difference. 2 h, 6 h, 12 h, and 24 h indicate 2, 6, 12, and 24 hours' time points, respectively. Data are shown as mean ± SD (*n* = 6 per group). ^*∗*^*p* < 0.05; ^*∗∗*^and ^*∗∗∗*^*p* < 0.01; ^*∗∗∗∗*^*p* < 0.0001.

**Figure 6:**
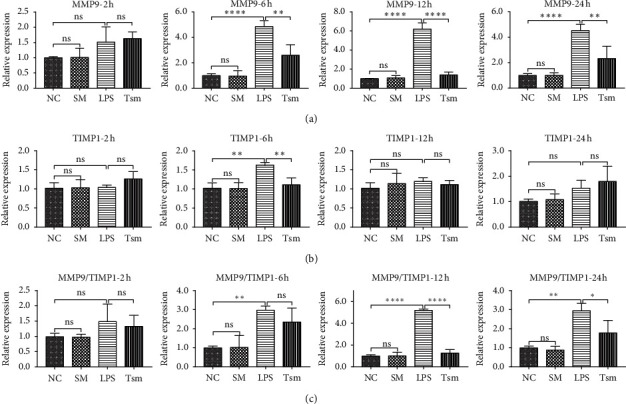
(a) MMP9 mRNA expression; (b) TIMP1 mRNA expression; (c) MMP9/TIMP1 ratio. NC, normal control group; SM, *Salvia miltiorrhiza* group; LPS, lipopolysaccharide group; Tsm, *Salvia miltiorrhiza* treatment group. ns, no statistical difference. 2 h, 6 h, 12 h, and 24 h indicate 2, 6, 12, and 24 hours' time points, respectively. Data are shown as mean ± SD (*n* = 6 per group). ^*∗*^*p* < 0.05; ^*∗∗*^and ^*∗∗∗*^*p* < 0.01; ^*∗∗∗∗*^*p* < 0.0001.

Compared with the NC group, the LPS group gradually increased expression of MMP2 and MMP9 mRNA with time: the changes in MMP2 were significantly different in the LPS group at all time points compared to the NC group (Figure 5(a)); the changes in MMP9 in the LPS group were significantly different compared to the NC group at the other three time points except for 2 hours (Figure 6(a)). The expression of TIMP1 and TIMP2 had also a slight increase in the LPS group compared to the NC group: the expression of TIMP2 had statistical differences at 6 and 12 hours (Figure 5(b)), whereas the expression of TIMP1 only had statistical difference at the 6 hours' time point (Figure 6(b)).

Compared with the LPS group, the Tsm group had a gradual reduction in the expression of MMP2 and MMP9 mRNA at 6, 12, and 24 hours' time points (Figures 5(a) and 6(a)); however, there was no statistical difference in the expression of TIMP1 and TIMP2, except for TIMP1 at 6 hours (Figures 5(b) and 6(b)).

The LPS group exhibited a gradually increased ratio of MMP2/TIMP2 and MMP9/TIMP1 with time compared to the NC group at the 6, 12, and 24 hours' time points. However, the ratio decreased gradually in the Tsm group until the balance was gradually restored. There was no significant difference in MMP2/TIMP2 and MMP9/TIMP1 ratio between the SM group and the NC group (Figures 5(c) and 6(c)).

**Figure 7:**
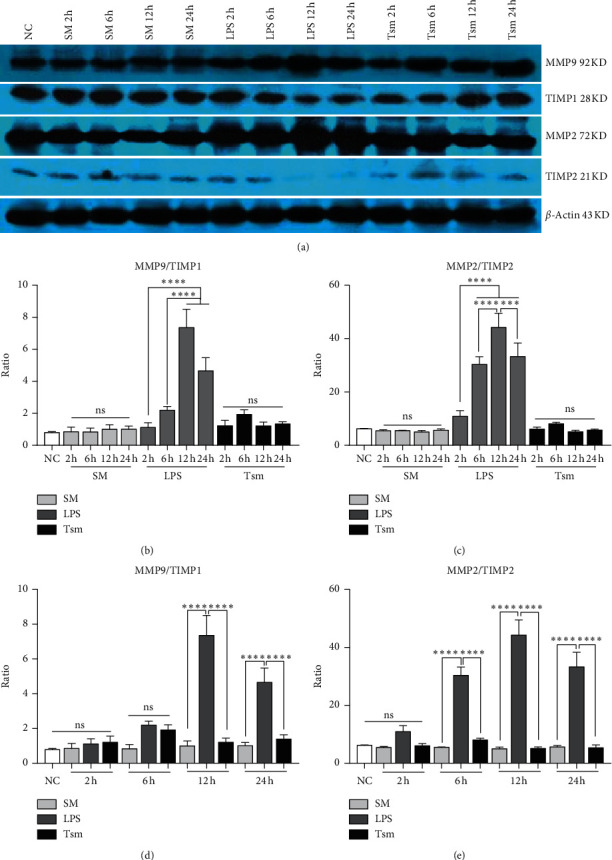
Lung MMPs and TIMPs protein expression. (a) Western blot electropherogram; (b) intragroup comparison of MMP9/TIMP1 ratio; (c) intragroup comparison of MMP2/TIMP2 ratio; (d) comparison of MMP9/TIMP1 ratio among groups; (e) comparison of MMP2/TIMP2 ratio among groups. NC, normal control group; SM, *Salvia miltiorrhiza* group; LPS, lipopolysaccharide group; Tsm, *Salvia miltiorrhiza* treatment group. ns, no statistical difference. 2 h, 6 h, 12 h, and 24 h indicate 2, 6, 12, and 24 hours' time points, respectively. Data are shown as mean ± SD (*n* = 6 per group). ^*∗∗∗*^*p* < 0.01; ^*∗∗∗∗*^*p* < 0.0001.

Protein expression of MMP2, TIMP2, MMP9, and TIMP1 and the changes of MMP2/TIMP2 and MMP9/TIMP1 ratio in each group were consistent with the change trend of mRNA level (Figure 7).

## 4. Discussion

ALI is a common disease in the critical care clinic. Due to its low cure rate and high fatality rate, survivors are often left with significant respiratory dysfunction [24, 25], but the pathogenesis has not yet been clarified. Studies have shown that Gram-negative bacterial infection can release LPS to induce inflammatory storms [26, 27]. A large number of cytokines, such as TNF-*α* and IL-6 release, lead to neutrophil aggregation in lung tissue, destruction of alveolar epithelial structure [28, 29], ECM damage, and alveolar-capillary membrane injury, thereby causing exudative pulmonary edema [10]. In the current study, after 6 hours after injection of LPS, the rats developed irritability, cyanosis, and shortness of breath. Serum inflammatory cytokines TNF-*α* and IL-6 increased with time. LPS injection is a known experimental model causing lung inflammation [30, 31]; in the current study we confirmed the detrimental effects up to 6 hours after LPS injection.

The ECM is the main structural component of the alveolar-capillary membrane, which not only plays a mechanical-supporting and mechanical-connecting role between cells but is also important in maintaining normal tissue structure and function [9, 32, 33]. Previous work has shown that ECM metabolic disorders are involved in the pathophysiological process of ALI and chronic lung injury [34, 35].

MMPs are proteolytic enzymes that rely on the metal ions zinc and calcium and use ECM components as hydrolysis substrates; they play an important role in maintaining the normal transformation of ECM, tissue damage and repair, inflammatory response, and other pathophysiological processes [11, 12, 36]. Thus far, more than 20 species of MMP family members have been identified. Among these members, MMP2 and MMP9 are known to degrade the main structural components of the basement membrane, such as type IV collagen. As such, they play an important role in the degradation of the alveolar-capillary membrane and the maintenance of the integrity of the basement membrane [37–39].

Among the TIMPs, TIMP1 is an MMP9-specific tissue inhibitor and TIMP2 is an MMP2-specific tissue inhibitor [40, 41]. The cysteine residues in the N-terminal domain of TIMPs bind to the zinc ion active center of MMPs to form MMP-TIMP complexes in a 1 : 1 ratio, thereby blocking the binding of MMPs to ECM substrates, effectively inhibiting the activities of MMPs and participating in the regulation of ECM synthesis and degradation [42, 43].

Under normal circumstances, the proportion of MMPs/TIMPs in the lung and the synthesis and degradation of ECM are in a state of dynamic equilibrium [44]. A disturbance in this balance may increase ECM degradation, leading to destruction of normal tissue structure and aggravation of lung injury. Conversely, if ECM degradation is reduced, ECM accumulated in the stroma may lead to fibrosis of the lung tissues [12, 45].

The results of the current study showed that expression levels of MMP2 and MMP9 mRNA and protein gradually increased with time in the LPS group, thus causing ECM damage in the alveolar-capillary membrane. In addition, the corresponding tissue inhibitors TIMP1 and TIMP2 were increased to varying degrees in the LPS group suggesting limited tissue repair. Also the ratio of MMP2/TIMP2 and MMP9/TIMP1 increased gradually with time in the LPS group; concomitantly, the lung W/D ratio and lung permeability index were increased significantly, indicating the occurrence of ALI.

*Salvia miltiorrhiza* injection (SMI), a Chinese Materia Medica standardized product extracted from the root of red-rooted Salviae Miltiorrhizae (*Salvia miltiorrhiza* Bunge, Labiatae, Danshen in Chinese), is an injectable solution through modern formulation and preparation process. The main ingredient is tanshinone, which plays an active role in the treatment of many diseases such as promoting blood circulation, removing blood stasis, reducing inflammatory reaction, and improving coagulation, as well as other effects [46, 47].

In this study, SMI one hour after LPS injection caused serum TNF-*α* and IL-6 in the Tsm group to significantly decrease, along with a reduced inflammatory reaction. Additionally, the lung W/D ratio and lung permeability index were decreased significantly, suggesting that *Salvia miltiorrhiza* reduced pulmonary edema. The pathological analysis showed that the degree of lung injury was gradually reduced over time, suggesting that SMI reduced lung injury. At the same time, we found that the expression of MMP2 and MMP9 in the Tsm group was gradually downregulated over time. The ratio of MMP2/TIMP2 and MMP9/TIMP1 decreased gradually in the Tsm group until the ratio gradually regained balance. This suggested that the protective effect of SMI on ALI might be mediated by rebalancing of MMPs/TIMPs ratio.

## 5. Conclusions

In summary, our data suggest that SMI can alleviate acute lung injury caused by LPS injection by restoring the balance of MMPs/TMPs ratio. This presents a new avenue of research to pursue clinical applications of this potential treatment.

## Figures and Tables

**Table 1:** Primer sequences for real-time quantitative PCR analyses.

Gene name	Forward primer	Reverse primer
MMP2	ACCGAGGATTATGACCGGGA	GCTGGTGCAGCTCTCATACT
MMP9	TCGGATGGTTATCGCTGGTG	AAGACGCACATCTCTCCTGC
TIMP1	ACAGCTTTCTGCAACTCGGA	CGGAAACCTGTGGCATTTCC
TIMP2	CGAGAAGGAGGTGGATTCCG	CCGCCTTCCCTGCAATTAGA
*β*-actin	TGTCACCAACTGGGACGATA	GGGGTGTTGAAGGTCTCAAA

MMP2, matrix metalloproteinase 2; MMP9, matrix metalloproteinase 9; TIMP1, tissue inhibitor of metalloproteinase 1; TIMP2, tissue inhibitor of metalloproteinase 2.

**Table 2:** Related antibodies and companies required in Western blot analysis.

Antibody name	Source company and catalog no.
Goat anti-rat MMP2	R&D Biotech Co., Ltd.; cat. no. AF1488-SP
Goat anti-rat TIMP1	R&D Biotech Co., Ltd.; cat. no. AF580-SP
mouse anti-rat *β*-actin	R&D Biotech Co., Ltd.; cat. no. MAB8929-SP
Mouse anti-rat MMP9	Novus Biologicals Inc.; cat. no. NBP2-13173SS
Mouse anti-rat TIMP2	Novus Biologicals Inc.; cat. no. NBP1-42375
Goat anti-mouse secondary antibodies	Boster Biological Technology Co., Ltd. (Wuhan, China); cat. no. BA1050
Rabbit anti-goat secondary antibodies	Boster Biological Technology Co., Ltd. (Wuhan, China); cat. no. BA1060

MMP2, matrix metalloproteinase 2; MMP9, matrix metalloproteinase 9; TIMP1, tissue inhibitor of metalloproteinase 1; TIMP2, tissue inhibitor of metalloproteinase 2.

## Data Availability

The TIF and JPG data used to support the findings of this study are available from the corresponding author upon request.
